# The Association between Pain-Related Variables, Emotional Factors, and Attentional Functioning following Mild Traumatic Brain Injury

**DOI:** 10.1155/2012/924692

**Published:** 2012-04-23

**Authors:** Michelle Beaupré, Élaine De Guise, Michelle McKerral

**Affiliations:** ^1^Centre for Interdisciplinary Research in Rehabilitation-Centre de Réadaptation Lucie-Bruneau and Department of Psychology, Université de Montréal, Montréal, QC, Canada H2H 2N8; ^2^TBI Program, Montreal General Hospital, McGill University Health Centre, Montréal, QC, Canada H3G 1A4

## Abstract

This study examined how MTBI concomitants such as pain variables, depression, and anxiety were related to attentional functioning at different stages of recovery. Participants having sustained a MTBI who were in the earlier phase of recovery showed, compared to controls, slower reaction times and larger intra-individual variability on a Computerized Pictorial Stroop Task (CPST). They also reported more post-concussion symptoms, pain intensity and disability, whereas MTBI participants who were in the later phase of recovery presented a higher rate of post-concussive symptoms and somewhat higher pain intensity/disability. MTBI participants' scores on the cognitive items of the post-concussion symptoms scale were positively correlated with reaction times on the CPST, while pain intensity/disability levels were negatively correlated with standard attention measures. Results indicate that obtaining response times and intra-individual variability measures using tests such as the CPST represents an effective means for measuring recovery of attentional function, and that pain intensity/disability should be systematically assessed after a MTBI.

## 1. Introduction

Individuals who have sustained a mild traumatic brain injury (MTBI) may manifest postconcussional symptoms of a physical, cognitive, or emotional nature [1, 2]. Possible cognitive symptoms include difficulties with concentration, attention, memory, executive functioning [3], word finding, and information processing [4]. Therefore, the cognitive impact of MTBI can be extensive and wide ranging [5]. It is well known that of the reported symptoms, attention is especially problematic for many individuals having had a MTBI. In fact, meta-analytical studies indicate that attention deficits are the most persistent neuropsychological complaint following closed-head injury [6, 7]. Understanding these attentional difficulties is important in planning management and rehabilitation of persons suffering from the consequences of MTBI.

Multiple studies have demonstrated that MTBI produces attentional deficits [8, 9]. In fact, there appears to be a large variety of attentional deficits found within this group of patients in terms of both reported symptoms and neuropsychological performance [5]. Divided attention deficits and sustained attention deficits have been identified in the MTBI population [10–12]. As well, results of some experimental studies suggest that MTBI may produce difficulty in effectively filtering relevant sensory information from irrelevant information, or of selective attention [9, 13].

Pathophysiologically, acceleration, deceleration, and rotation forces are involved in traumatically induced brain injuries [14]. These forces cause microscopic shearing and diffuse damage to neurones [14, 15] in the cortex as well as in subcortical white-matter. As well, these forces also lead to a complex neurometabolic/neurochemical cascade that interferes with axonal transport [16, 17] and can result in axonal blebbing and eventual disconnection [18]. Certain areas of the brain are more likely to be impacted by such processes, particularly the frontal lobes [15, 19]. The frontal cortex accounts for executive functioning, attention processes, working memory, and self-regulation [20]. Hence, deficits of attention identified following a MTBI are compatible with the pathophysiological changes that may occur with this type of injury.

Postconcussional symptoms usually resolve within 3 months [21]. Nonetheless, in approximately 5% to 15% of cases, individuals continue to show symptoms (including cognitive and attentional complaints) that are sometimes but not always identified on neuropsychological testing [2]. Of the many variables that are reportedly associated with persisting symptomatology are female gender, older age, poor academic achievement, lack of social support, and previous head injury [22]. Psychological factors have also been associated with persisting symptomatology and specifically, with persisting cognitive symptoms [23]. For example, Sherman et al. [24] found evidence that the depression status of persons having sustained a mild head injury 2 years before was related to scores on visual attention tasks and on psychomotor measures. Also, Gass [25] found that closed-head injury patients at 2.6 years after injury obtained scores on measures of attention that were related to scores obtained on the MMPI-2 anxiety scale. It is unclear if these associations between depression symptoms, anxiety symptoms, and performance on attention tasks are also present in the earlier stages of recovery that follow MTBI.

In trying to understand the attentional complaints of those having suffered a MTBI, one must consider the fact that acute pain and chronic pain problems may also be involved. It is in fact quite common for those having had a head injury to also present with comorbid pain issues. A study examining the prevalence of chronic pain (i.e., still present after 3 months) following TBI found that it was a very common complication [26]. It is especially common in the MTBI population, as indicated by the findings of Uomoto and Esselman [27], who reported that 95% of individuals that had sustained a MTBI reported chronic pain while only 22% of those having suffered a moderate- to-severe TBI did so. As for the type of pain presented by patients, Guérin et al. [28] found that 70% of individuals receiving out-patient holistic rehabilitation services following a MTBI presented posttraumatic headaches, while over 85% reported significant pain symptomatology in other parts of the body.

Chronic pain in itself is known to impact cognitive functioning. A review examining the impact of chronic pain on neuropsychological functioning [29] found that impairments on measures of attention, processing speed, and psychomotor speed were often present in the chronic pain population. There has also been some interest for the study of the stimulus-driven effects of pain-related information, on the attention of pain patients. Some research groups have in fact found evidence, using modified Stroop tasks and dot-probe paradigms [30] that chronic pain patients may develop a hypervigilance to pain, pain-associated information and environmental stimuli representative of pain, hence, having difficulty disengaging from this type of information and manifesting an attentional bias for this type of information [31]. Consequently, pain may compete with other attentional demands, leading to difficulties with attention [32].

There has been little research examining the cognitive/neuropsychological performance of TBI patients suffering from comorbid pain, as many studies have excluded this group that presents potential confounds. Furthermore, the existing studies have often specifically examined the effect of head and neck pain, which is frequent in the TBI population, but has also been suggested as being a particular type of pain which may increase vulnerability to cognitive impairment [29]. There are no published articles that have examined the impact of nonheadache/neck pain on the cognitive functioning and more specifically on attentional function of persons having sustained a MTBI, but some information may be provided by taking a closer look at a study performed with TBI patients with injuries of a variety of severity levels. In an abstract, Vernon-Wilkinson and Tuokko [33] describe how they examined the records of patients referred for assessment of head injury. Records were divided into those with and those without pain, regardless of the severity of brain injury. Although the brain injured patients with comorbid pain had less severe head injuries in terms of Glasgow Coma Scale scores as well as in terms of duration of posttraumatic amnesia and measures of ventricular enlargement, they did poorer than the brain injured patients without pain on many neuropsychological tests.

Hence, the assessment of attention following a MTBI can be influenced by a number of comorbidities, such as pain, symptoms of depression, as well as symptoms of anxiety. Each of these is frequently present in those having sustained a MTBI [26, 34, 35], may impact results obtained on measures of attention, and may also complicate or delay recovery, and must be better understood in order to provide appropriate rehabilitation interventions. The aim of the present study was to examine selective attention using both established neuropsychological measures and a newly designed Computerized Pictorial Stroop Task and to identify associations with MTBI concomitants using measures of depression, anxiety, pain variables, and postconcussive symptoms, in individuals having suffered a MTBI and who were in different stages of recovery (at 1–3 months after injury and at 5–7 months post-injury). Our hypotheses were that persons with MTBI perform worse than normal controls on selective attention measures, and that more important pain-related, cognitive and affective symptomatology is related to worse attentional performance, irrespective of stage of recovery.

## 2. Methods

### 2.1. Participants

Our study involved three groups of participants. These included two groups of individuals who had sustained a MTBI: 15 who were in the earlier phase of recovery, MTBI-early (mean months after injury = 2.2 ± 0.5; 10 men and 5 women, mean years of age: 39 ± 13, mean years of education: 13 ± 3), and 15 who were in the later phase of recovery, MTBI-late (i.e., mean months after injury = 5.6 ± 1.2; 13 men and 2 women, mean years of age: 38 ± 13, mean years of education: 13 ± 3). The third group (normal controls) was made up of 17 neurologically unimpaired adults (10 men and 7 women, mean years of age: 31 ± 11, mean years of education: 14 ± 2). One-way ANOVAs showed that there were no significant differences between the three groups for age (*F*(2,44) = 2.29, *P* = .11) and years of education (*F*(2,44) = 0.99, *P* = .38). Normal controls were recruited from the community through local advertisement. Some patient participants were recruited from a neurotrauma unit and the majority were from an out-patient intervention program offered in a major rehabilitation center. All subjects were French-speaking and they all provided written informed consent. The study was approved by the institutional ethical review board.

All participants in the MTBI groups had sustained a MTBI based on the criteria of the American Congress of Rehabilitation Medicine Brain Injury-Interdisciplinary Special Interest Group [36]. According to these criteria, MTBI results from traumatically induced physiological disruption of brain function, manifested by *at least one* of the following: (1) any period of loss of consciousness; (2) any loss of memory for events immediately before or after the accident; (3) any alteration of mental state at time of the accident (e.g., feeling dazed, disoriented, or confused); (4) focal neurologic deficit(s) that may or may not be transient; but where the severity of the injury does not exceed a loss of consciousness of approximately 30 minutes or less; after 30 minutes, an initial Glasgow Coma Scale of 13–15, and posttraumatic amnesia not greater than 24 hours. TBI severity was identified in patient medical files and was confirmed by reviewing medical records related to neurological indices and via comprehensive retrospective patient interviews. The majority of participants had uncomplicated MTBI, that is, had negative clinical brain imaging (complicated MTBI: 3 in the early group; 2 in the late group). The causes of injury in the MTBI-early group were motor vehicle accidents (*n* = 9), falls (*n* = 3), and physical assaults (*n* = 3). In the MTBI-late group, causes of injury were motor vehicle accidents (*n* = 8), falls (*n* = 5), physical assault (*n* = 1), and a work-related accident (*n* = 1). Potential participants were excluded from the study for the following reasons: previous TBI (except that for which MTBI participants were referred), headache or neck pain, alcohol or substance abuse within 6 months prior to testing, intake of medication which can affect cognition (ex. opioids, anticonvulsants), or uncorrected visual impairment.

### 2.2. Materials

#### 2.2.1. Self-Report Questionnaires

**(1) French Version of the Beck Depression Inventory: Second Edition**

The Beck Depression Inventory-II [37] is a 21-item questionnaire that is used to assess symptoms of depression. Each item is rated on a Likert scale ranging from 0 to 3. A score of <10 means no or minimal depression, 10 to 17 is mild to moderate depression, and 18 to 29 is moderate to severe depression, and severe depression is from 30 to 63.

**(2) French Version of the Beck Anxiety Inventory**

The Beck Anxiety Inventory [38] is made up of 21 items, each corresponding to a symptom that presents in anxiety disorders. Respondents must rate each symptom on a scale ranging from 0 to 3, based on how frequently they have suffered from that symptom in the past 7 days. A score of <7 represents a minimal level of anxiety, 8–15 represents mild anxiety, 16–25 is interpreted as moderate anxiety, and 26–63 represents severe anxiety.

**(3) French Version of the Postconcussion Scale**

The Postconcussion Scale [39] questionnaire was filled out by all participants. This scale was developed to provide a formal method of documenting postconcussion symptoms [40]. The scale is made up of 22 commonly reported physical, cognitive, and affective symptoms of TBI (e.g., dizziness, difficulty concentrating, sadness) and is commonly used with the MTBI population. Patients are asked to rate the intensity of every symptom on a Likert scale ranging from 0 to 6.

**(4) Present Pain Intensity from the French Version of the McGill Pain Questionnaire**

The Present Pain Intensity of the McGill Pain Questionnaire [41] is a ten-word intensity rating scale. Pain is rated from 0, which signifies “no pain,” to 10, which is “intolerable” or “excruciating.”

**(5) French-Canadian Version of the Pain Disability Index**

The Pain Disability Index [42] is designed to help patients measure the degree to which their daily lives are disrupted by persistent pain. It is made up of 7 items (each corresponds to a category of life activity, e.g., recreation, occupation) that the patient must rate on a Likert scale ranging from 0 to 10. For each of the categories, a score of 0 means no disability at all, and a score of 10 is equivalent to complete disability.

All questionnaires are widely used and both their English and French versions have been found to have excellent validity and reliability.

#### 2.2.2. Assessment of Attention

**(1) Map Search from the Test of Everyday Attention (TEA) [43]**

Participants each had 2 minutes to identify as many target stimuli as they could on a map. The obtained score was the number of targets identified out of a maximum of 80. Raw scores were used for statistical analyses.

**(2) Telephone Search from the TEA [43]**

Participants had to look for designated key symbols and ignore other symbols, while searching entries in a simulated classified telephone directory. The score was calculated by dividing the total time taken to complete the search, by the number of symbols detected. The maximum number of symbols that could be detected was 20. Raw scores were used for statistical analyses.

**(3) Ruff 2 and 7 [44]**

Participants completed the 20 trials (10 letter trials and 10 digit trials) of this visual search and cancellation task. In each of the trials, participants were required to detect and mark through as many as possible of the two target digits: “2” and “7,” within a 15 s time limit. Automatic Detection Accuracy raw score and Controlled Search Accuracy raw score were used for statistical analyses. Automatic Detection Accuracy raw score is calculated by dividing the Automatic Detection Speed raw score by the sum of the Automatic Detection speed and Error raw scores and by multiplying this number by 100. Controlled Search Accuracy is calculated by dividing the Controlled Search Speed raw score by the sum of the Controlled Search Speed and Controlled Search Errors raw scores and by multiplying this number by 100.

**(4) Computerized Pictorial Stroop Task**

The Computerized Pictorial Stroop Task is a selective attention task which involves a patient/participant naming the colour of the frame surrounding a picture, as quickly as possible. Hence, the patient must process the relevant information (colour of the frame) while trying to filter the irrelevant information (the picture). The development of this task stems from studies utilizing the modified Stroop paradigm, which found evidence that chronic pain patients selectively attend to both sensory and affective pain words (in comparison to neutral words) [45]. It was thought that replacing the words by pictures would add ecological validity to the task, as this aspect of the task had before been criticized [46, 47].

The task involved participants completing 2 blocs comprised of 80 trials (or pictures) each. The task included three categories of pictures: pictures representing pain, pictures representing anger, and neutral pictures. Pain pictures consisted of pictures of human faces expressing pain, and pictures of hands and feet in pain-evoking situations stemming from previously published picture databases in which the intensity of emotion was considered and controlled during the development of the database [48, 49]. Pictures representative of anger consisted of pictures of human faces presenting facial expressions of anger, and stemmed from the same database as pictures of human faces presenting expressions of pain [48]. Each of the experimental pictures was matched with a neutral picture (i.e., the same human face presenting a neutral facial expression or hand/foot in a non-pain-evoking situation). Pictures measured 256 × 256 pixels. Frames surrounding the presented pictures measured 8 pixels and the picture-frame distance varied in random fashion. Frames were presented in each of the following four equiluminant colours: red, yellow, green, and blue. The stimuli were presented in the center of the screen of a laptop computer. Patients were instructed to say out loud the colour names of each frame surrounding the presented pictures, as quickly as possible, without sacrificing accuracy. Latency in naming the colour of the frame was recorded with a Plantronics USB DSP V2 microphone. Stimuli were presented for 1000 ms and the interstimulus interval varied between 500–750 ms.

Measures obtained from the Computerized Pictorial Stroop Task included mean reaction time and intraindividual variability of reaction time. Mean reaction time was calculated by adding a particular patient's reaction times to all trials involving a certain type of picture (pain, anger, or neutral). This total was then divided by the number or trials that were summed up. The intraindividual variability of reaction time was calculated by computing the difference between the reaction time to each of the trials involving a certain type of picture (either pain, anger, neutral) and the mean reaction time to that type of picture. Each of these differences from the mean was then squared and the average of these values was calculated. The square root of this average is the intraindividual variability of reaction time. Examining intraindividual standard deviation as a measure of variability/consistency of performance has been used in prior head injury studies [50].

### 2.3. Procedure

All subjects participated in an individual testing session. They first underwent a screening of medical and psychiatric history, which included questions regarding pre- and postmorbid functioning, self-reported symptomatology, and psychiatric history. Following this, anxiety, depression, pain disability, and postconcussion symptoms as well as current pain intensity were assessed by means of the self-report questionnaires. Patients were randomly assigned to first complete either the standardized attention tasks, or the Computerized Pictorial Stroop Task. During the Computerized Pictorial Stroop Task, patients were seated at a distance of 57 cm from the computer screen and they were asked to focus on the centre of the screen at all times. Patients practised the colour-naming task with 10 pictures, which were not included in the experimental set, until they understood the task. The pictures were presented in a random sequence.

### 2.4. Statistical Analyses

Descriptive statistics were first computed to determine group means and standard deviations obtained on the standardized attention tests, on the Computerized Pictorial Stroop Task and on self-report inventories. Three separate univariate ANOVAs using planned contrasts (MTBI-early versus normal controls; MTBI-late versus normal controls; MTBI-early versus MTBI-late) were then calculated for the TEA subtests (Telephone Search and Map Search) and the Ruff 2 and 7 Selective Attention Test, in order to determine if there were differences in results between the three groups on these measures. Two-way repeated-measures ANOVAs, with image type as the within-subject factor (3 levels: pain, anger, neutral) and with group as the between-subject factor, were used to determine whether there were differences in reaction times, intraindividual standard deviation of reaction times and error rates for each condition of the Computerized Pictorial Stroop Task. Univariate ANOVAs using planned contrasts were then run to examine potential differences between the three groups on scores obtained on self-report questionnaires assessing pain, pain-related impairment, depression, anxiety, and TBI-related symptomatology. A series of Pearson correlations were then computed between scores obtained by participants with MTBI on each of the self-report inventories, and performance on the standardized measures of attention and the Computerized Pictorial Stroop Task conditions. Population variances were systematically assessed using Levene's test and variances were found to be homogenous in all cases. All data were analysed using SPSS version 17.0. The level used for statistical significance was  .05.

## 3. Results

### 3.1. Attentional Tasks (Map Search from the TEA, Telephone Search from the TEA and Ruff 2 and 7 Selective Attention Test)

Mean scores and standard deviations obtained by the three groups (MTBI-early, MTBI-late, and normal controls) on the standardized attentional tests are found in Table 1. According to the TEA manual, all three groups obtained scores that fell within the normal-range (i.e., above the clinical cut-off) on the Map Search and the Telephone Search. Separate three-group ANOVAs revealed that the groups did not differ significantly in their performance on the Telephone Search (*F*(2,44) = 1.53, *P* = .23) nor on the Map Search (*F*(2,44) = 1.42, *P* = .25). As well, separate three-group ANOVAs revealed that the groups did not differ significantly in their performance on the Ruff 2 and 7 Selective Attention Test, on both automatic detection accuracy (*F*(2,44) = 2.62, *P* = .08) and controlled search accuracy (*F*(2,44) = 2.25, *P* = .12).

### 3.2. Computerized Pictorial Stroop Task

**Generalized Slowing**

Mean reaction times to images (pain, anger, neutral) of the three groups (MTBI-early, MTBI-late, normal controls), as well as their respective standard deviations, are found in Table 2. The repeated-measures ANOVA indicated no significant interaction between image type and group (*F*(4,88) = 1.41, *P* = .24). It did yield a significant group effect (*F*(2,44) = 4.09, *P* = .02). Post hoc Tukey's tests showed that the MTBI-early group had significantly slower reaction times than those of normal controls, as well as those of the MTBI-late group. All other comparisons were not significant. For the impact of image type (pain, anger, neutral) on reaction time, there was no difference among the different conditions (*F*(2,88) = .016, *P* = .98).

**Variability in Response Times between Trials**

Intraindividual standard deviation of reaction times to images (pain, anger, neutral) obtained by the three groups are found in Table 3, along with the standard deviations for each group. The two-way repeated measures ANOVA performed on the intraindividual standard deviation of response times showed no significant interaction between image type and group (*F*(4,88) = 1.05, *P* = .39). It did yield a significant main effect of group (*F*(2,44) = 3.49, *P* = .04). Post hoc tests demonstrated that individuals of the MTBI-early group had significantly more variable response times than did normal controls. Other comparisons were not significant.

The ANOVA also revealed a significant effect of image type on intraindividual variability of response time amongst trials (*F*(2,88) = 11.87, *P* = .00). All of three pairwise comparisons that were conducted were significant at the  .05 level. These demonstrated that intraindividual variability in response times for images of pain was significantly greater than that of images of anger and of that of neutral images. In addition, variability in response times for neutral images was significantly greater than that of images of anger.

**Errors**

The ANOVA indicated no significant interaction between image type and group (*F*(4,88) = 0.70, *P* = .59). As well, no main effect of group was identified (*F*(2,44) = 2.32, *P* = .11), nor was there a main effect of image type on errors made (*F*(2,88) = 2.60, *P* = .08).

### 3.3. Self-Report Questionnaires Assessing Depression, Anxiety, TBI-Related Symptomatology, Pain, and Impairment

**BDI-II and BAI**

Scores obtained by the three groups on the self-report questionnaires can be found in Table 4. One-way ANOVAs did not reveal any significant group effects on scores obtained on the Beck Depression Inventory-II and on the Beck Anxiety Inventory (*F*(2,44) = 1.67, *P* = .20 and *F*(2,44) = 0.47, *P* = .63, resp.).

**TBI-Related Symptomatology**

A one-way ANOVA found the effect of group on the PostConcussion Scale to be significant (*F*(2,44) = 6.35, *P* = .00). Posthoc Tukey's tests showed that the both MTBI-early and MTBI-late groups obtained significantly higher scores on the self-report inventory than did normal controls. All other comparisons were not significant.

**Pain Intensity**

A one-way ANOVA determined that the effect of group on the Present Pain Intensity Scale of the McGill Pain Questionnaire was significant (*F*(2,44) = 4.29, *P* = .02). Pos thoc Tukey's tests found that the MTBI-early group endorsed significantly higher levels of pain than did normal controls. The MTBI-late group also showed higher pain intensity levels than to controls, but the difference did not reach significance.

**Disability Associated with Pain**

A one-way ANOVA was computed and found the effect of group on the Pain Disability Index to be significant (*F*(2,44) = 6.94, *P* = .00). Post hoc Tukey's tests revealed that the MTBI-early group obtained significantly greater scores on the self-report inventory than did normal controls. Again, the MTBI-late group presented higher pain disability scores than controls, but the difference did not reach significance.

### 3.4. Correlations between Self-Report Questionnaires and Performance

In order to determine if performance was modulated by effect and by pain symptomatology and disability in the MTBI population, we computed Pearson correlations between scores obtained by all MTBI participants on each of the four self-report inventories (BDI-II, BAI, Present Pain Intensity Scale, Pain Disability Index), and results they obtained on both standard attention measures and the Computerized Pictorial Stroop Task. The correlation between scores obtained by participants with MTBI on the Present Pain Intensity Scale and scores obtained on the Map Search was significant (*r*(28) = −.56, *P* = .001), as was that between scores obtained by MTBI participants on the Present Pain Intensity Scale and those obtained on the Telephone Search (*r*(28) = −.39, *P* = .03). As well, the correlation between scores obtained by participants with MTBI on the Pain Disability Index and scores obtained on the Map Search was significant (*r*(28) = −.4, *P* = .03). No other correlation reached significance.

In order to evaluate if performance was modulated by TBI-related cognitive symptoms, we computed Pearson correlations between scores obtained by MTBI participants on the cognitive items of the Postconcussion Scale and results they obtained on both standard attention tasks and the Computerized Pictorial Stroop Task. There were significant correlations between this cognitive component of the Post-Concussion Scale and response times to images of pain (*r*(28) = .56, *P* = .00), anger (*r*(28) = .5, *P* = .005), and neutral images (*r*(28) = .52, *P* = .003). None of the correlations between intraindividual variability in response times and the cognitive component of the Post-Concussion Scale were significant. Also, correlations between standard tests of attention and the cognitive component of the Post-Concussion Scale did not reach the significance level.

## 4. Discussion

Findings from the present study indicate that individuals having had a MTBI may manifest reaction time deficits on a selective attention task. In fact, MTBI participants in earlier phases of recovery showed both slower reaction times as well as less consistent reaction times than healthy normal controls on the Computerized Pictorial Stroop Task. These findings are consistent with prior literature which has consistently revealed a generalized slowing of information processing following head injury [51]. Many authors have suggested that this deficit in information processing is related to diffuse axonal injury (DAI), which typically occurs with TBI [52]. Although to a lesser extent than in more severe TBI, diffuse injury to white matter tracts following MTBI could presumably reduce interconnections between neural networks, thereby reducing the speed at which information is transmitted.

The observed disturbance in the intraindividual variability in reaction time of individuals having sustained a MTBI and who were in the earlier phases of recovery is also consistent with results of previous studies. It has been observed for many years that brain damage causes increased intraindividual variability [53]. Numerous studies using various methods for measuring variability have in fact confirmed greater variability in patients with TBI at all levels of severity, with or without focal frontal lesions [50, 51, 54–57]. In TBI, the extent of intraindividual variability is closely associated with impaired maintenance of stable “top-down” attentional processes, where large intraindividual variability can be due to a general deficit in regulation of attention in any cognitive domain, and is most consistently described in patients with impairments in executive functions [53].

Our findings did not indicate a general slowing of responses (or attentional bias) specific to pain pictures; however we did obtain a main effect of image type on intraindividual variability. Potential explanations of this effect may lie in literature examining the impact of emotional stimuli on attention. In fact, it is well documented that emotional stimuli can interfere with ongoing activities [58]. While several theories exist as to how and why effect influences attention [59], the arousal theory has received much interest. According to this theory, responses to affective pictures vary with the intensity of emotion evoked by the picture. Pictures found to be more arousing, command more attention and hence can interfere with ongoing tasks [59]. Since the pictures used in this study were controlled for emotional intensity, it is likely that the higher intraindividual inconsistency of responses found for images of pain reflected an increased attentional load, resulting in the observed variability for this type of picture. The main effect found, where pain pictures produced more variable responses overall compared to other picture types, but not specifically in the MTBI groups, suggests that our study patients did not show a higher attentional bias toward pain pictures. This is interesting in light of the fact that pain intensity and pain-related disability ratings also were not related to MTBI participants' performance on the Computerized Pictorial Stroop Task, contrary to standard measures of attention, suggesting that this task was resistant to pain-related variables.

Notwithstanding the above and noteworthy is the fact that these deficits in the speed and variability of reaction time were only apparent in the MTBI group in earlier phases of recovery and not in the MTBI group in later phases of recovery. This result corroborates previous findings that indicate that persons that sustain a MTBI generally recover in the 3-month period that follows their injury in terms of their ability to perform adequately on cognitive/neuropsychological measures [4, 60, 61]. Prior studies have also shown that the post-MTBI focal parenchymal lesions seen on MRI brain scans resolve within 1 to 3 months following injury and that these changes are paralleled by improvements on neuropsychological tests [60]. These last results likely indicate some parallel between neurological recovery, and recovery as seen on cognitive/neuropsychological measures. Important to mention, however, is that while objectively quantified results in our study indicated cognitive improvement in persons having had a MTBI who were in later phases of recovery, this was not reflective of subjective symptoms or of subjectively perceived status, as measured by the Post-Concussion Scale. In fact, MTBI participants in later phases of recovery also were reporting significantly greater amounts of symptoms than normal controls. As will be discussed in later paragraphs, existing literature supports the fact that the improvement of cognitive functions (assessed via neuropsychological measures) and subjective recovery (assessed via self-report symptom scales such as the Post-Concussion Scale) do not always follow the same course, with cognitive recovery either preceding or following the resolution of subjective symptoms [62].

In contrast to the above finding that participants with MTBI who were in earlier phases of recovery show impairment on a computerized reaction time task, analysis of results obtained by MTBI participants on conventional neuropsychological tests of selective attention did not show any impairment compared to normal controls. This is in line with existing evidence that reaction time procedures can reveal cognitive impairment even when normal performance is shown on traditional neuropsychological measures [51]. In fact, conventional neuropsychological tests were designed initially to detect quite severe impairments in patients with neurological and psychiatric illness, in patients with brain lesions and in people exposed to neurotoxic substances [63]. Detection of more subtle cognitive changes or the sensitivity of a particular measure is obviously of particular importance when conducting the neuropsychological assessment of persons who sustain a MTBI. A number of studies have shown the utility of computerized reaction time measures in detecting cognitive changes associated with MTBI [54]. This supports the recommendation made by several authors that clinical neuropsychological evaluations comprise reaction time measures [64]. There are in fact many advantages (both theoretical and practical) to computerized reaction time testing [63]. Among these is the fact that relatively high test-retest reliability coefficients and split half coefficients are reported for RT tests [65, 66]. As well, RT measures are not only useful in initial assessments of cognitive functioning following injury, but also as a tool to track its recovery, as they lack practice effects [67].

MTBI participants in earlier as well as in later phases of recovery were found to be reporting significantly greater levels of postconcussive symptoms than normal controls. This is in agreement with existing literature suggesting that long beyond the typical recovery (or the typical period of resolution of symptoms) interval of 1 to 3 months, it is common for persons having suffered a MTBI to report persisting difficulties [2, 68, 69]. Nonetheless, this finding does raise questions about the discrepancy between objective neuropsychological/neurobehavioural indicators and subjectively reported symptomatology. In fact, in our study, significant subjective symptoms were identified by the MTBI group in later phases of recovery, while no impairment was found in this group on any neuropsychological measure, including the Computerized Pictorial Stroop task. Possible explanations of this discrepancy may be found in the correlations between the Post-Concussion Scale and performance on attention measures. Specifically, we found no correlation between the Post-Concussion Scale total score and performance on standard tests of attention. We did however find that the total score obtained on cognitive items of the scale was significantly correlated to reaction times on the Computerized Pictorial Stroop Task. This finding seemingly illustrates that the self-report symptom scale used in this study assesses a variety of symptoms commonly reported by persons having had a MTBI (e.g., physical/somatic, psychological, and cognitive symptoms) and hence its total score provides a more global picture of recovery that is not restricted to the cognitive domain. Individuals that sustain a MTBI may thus continue to present a variety of symptoms in later phases of recovery (as seen on the self-report symptom scale), reflecting general functional recovery. In fact, as an alternative to mean group results where it is often impossible to appreciate varying levels of clinical evolution, individual cognitive recovery could be best gauged by examining scores obtained on the Computerized Pictorial Stroop Task, a reaction time measure, which was associated with the cognitive score of the PostConcussion Scale.

Results of our study indicated that participants with MTBI who were in earlier phases of recovery had significantly greater levels of pain (other than head/neck pain) than normal controls at the time of assessment and also presented with significantly greater levels of disability associated with their pain in everyday life. The MTBI-late group also showed higher, although not significantly, pain intensity and pain disability levels compared to controls, indicating that the pain-related recovery process was probably not attained around 6 months after injury. This is not surprising as literature provides evidence of high rates of comorbid pain in the TBI population [26]. As to understand how this reported pain may have impacted performance on attention tasks, we looked to the results of correlations computed between pain questionnaires and attentional performance. Interestingly, mirroring depression and anxiety results, pain and disability levels were not related to performance on our reaction time measure, the Computerized Pictorial Stroop Task, but they did modulate performance on certain standardized neuropsychological measures of attention, while depression and anxiety scores did not. Pain intensity, at the time of the assessment, modulated performance on the Map Search and the Telephone Search of the TEA, while pain disability was linked to results on the Map Search. These findings indicate that even in the absence of head/neck pain, the level of other types of pain at the time of the assessment as well as pain disability should be considered and assessed systematically following MTBI, as they may impact performance on standardized attention tasks. This is consistent with results of studies indicating that pain may affect scores obtained on neuropsychological measures of attention [29]. However, the fact that these variables did not affect response times on the Computerized Pictorial Stroop Task suggests that this measure is robust and much less susceptible to the effects of psychological and pain-related variables. The latter points are particularly important in regards to providing adequate and individually-designed rehabilitation interventions to individuals within this clinical population.

Possible limitations of this study must be considered. This investigation involved a cross-sectional design and it is possible that a longitudinal design might have produced a slightly different representation of the relationship between neuropsychological performance and various psychological variables. In addition, the groups in our study were relatively small and results of this study should be replicated with larger sample sizes.

In conclusion, our results confirmed our study hypothesis and highlight the complex multifactorial nature of post-MTBI symptoms and deficits. Obtaining response times and intraindividual variability measures using sensitive tests such as the Computerized Pictorial Stroop Task described in the present study represent effective means for measuring attentional and cognitive recovery after a MTBI. Such tasks and metrics are more sensitive to subtle cognitive changes and less affected by pain-related variables than conventional neuropsychological tests of selective attention. Furthermore, since pain intensity and pain-associated disability can modulate performance on standard tests of attention, these variables should be systematically assessed in individuals having sustained a MTBI and related to results on standardized neuropsychological tasks to allow for their correct interpretation. These considerations can have a fundamental impact on the clinical evaluation, followup, and provision of adequate rehabilitation interventions for these patients.

## Figures and Tables

**Table 1:** Scores (mean ± SD) of the three groups (MTBI-early, MTBI-late, normal controls) on standardized measures of attention. *F* values and *P* values are given.

	Controls	MTBI-early	MTBI-late	Statistic	*P* value
Map search	77.53 (5.49)	72.53 (10.27)	73.87 (9.98)	*F* = 1.42	.22
Telephone search	2.44 (0.67)	2.88 (0.77)	2.94 (1.14)	*F* = 1.53	.23
Automatic detection accuracy	95.14 (3.51)	97.40 (2.62)	95.45 (2.55)	*F* = 2.62	.08
Controlled search accuracy	89.03 (7.60)	92.87 (4.51)	93.37 (3.53)	*F* = 2.25	.12

**Table 2:** Mean reaction times (in milliseconds) to images (pain, anger, neutral) for the three groups (MTBI-early, MTBI-late, normal controls). Standard deviations are in parentheses.

	Controls	MTBI-early	MTBI-late
Pain RT	475.3 (123.75)	620.0 (122.65)	478.7 (159.86)
Anger RT	489.4 (148.34)	596.7 (130.86)	486.0 (186.81)
Neutral RT	492.9 (131.42)	608.0 (111.18)	475.3 (161.24)

**Table 3:** Intraindividual standard deviation of reaction times (in milliseconds) to images (pain, anger, neutral) of the three groups (MTBI-early, MTBI-late, normal controls). Standard deviations are in parentheses.

	Controls	MTBIs-early	MTBIs-late
Pain	136.5 (43.87)	174.7 (41.90)	128.0 (42.63)
Anger	105.3 (61.45)	125.3 (28.00)	111.3 (55.14)
Neutral	115.9 (42.29)	148.7 (44.38)	122.7 (27.38)

**Table 4:** Scores (mean ± SD) of the three groups (MTBI-early, MTBI-late, normal controls) on self-report questionnaires. *F* values and *P* values are given.

	Controls	MTBI-early	MTBI-late	Statistic	*P* value
BDI	5.29 (±4.93)	9.20 (±6.06)	9.53 (±10.27)	*F* = 1.67	.200
BAI	5.94 (±4.28)	7.80 (±6.81)	9.33 (±15.61)	*F* = 0.47	.630
ESPC-R	2.18 (±4.45)	20.80 (±14.49)	20.93 (±26.55)	*F* = 6.35	.004
Present pain Intensity	0.94 (±2.11)	3.40 (±3.02)	2.47 (±2.00)	*F* = 4.29	.020
Pain disability	1.47 (±4.00)	20.40 (±17.13)	12.07 (±18.49)	*F* = 6.94	.002
